# The Ubiquitination of NF-κB Subunits in the Control of Transcription

**DOI:** 10.3390/cells5020023

**Published:** 2016-05-12

**Authors:** Patricia E. Collins, Izaskun Mitxitorena, Ruaidhrí J. Carmody

**Affiliations:** Centre for Immunobiology, Institute of Infection, Immunity and Inflammation, College of Medicine, Veterinary and Life Sciences, University of Glasgow, Glasgow G12 8TA, UK; i.mitxitorena.1@research.gla.ac.uk (I.M.); ruaidhri.carmody@glasgow.ac.uk (R.J.C.)

**Keywords:** ubiquitination, NF-κB, transcription

## Abstract

Nuclear factor (NF)-κB has evolved as a latent, inducible family of transcription factors fundamental in the control of the inflammatory response. The transcription of hundreds of genes involved in inflammation and immune homeostasis require NF-κB, necessitating the need for its strict control. The inducible ubiquitination and proteasomal degradation of the cytoplasmic inhibitor of κB (IκB) proteins promotes the nuclear translocation and transcriptional activity of NF-κB. More recently, an additional role for ubiquitination in the regulation of NF-κB activity has been identified. In this case, the ubiquitination and degradation of the NF-κB subunits themselves plays a critical role in the termination of NF-κB activity and the associated transcriptional response. While there is still much to discover, a number of NF-κB ubiquitin ligases and deubiquitinases have now been identified which coordinate to regulate the NF-κB transcriptional response. This review will focus the regulation of NF-κB subunits by ubiquitination, the key regulatory components and their impact on NF-κB directed transcription.

## 1. Introduction

A little over 40 years ago, in the search for thymopoietin, Gideon Goldstein and colleagues isolated a small highly conserved 8.5 kDa polypeptide from bovine thymus [1,2]. Named after its ubiquitous expression pattern, ubiquitin is found in all eukaryotes and has been conserved over millions of years. Remarkably, human and yeast ubiquitin share just over 96% sequence identity and this evolutionary conservation emphasises the essential role ubiquitin plays in almost every biological process. However, the fundamental role of ubiquitin was not elucidated until 1980, when two studies outlined a mechanism of ATP-dependent proteolysis and the covalent linkage of ubiquitin to a protein substrate [3,4,5]. These pioneering studies outlined the basics of what is now known as the ubiquitin-proteasome system (UPS) and, unsurprisingly, led to the award of the Nobel prize in Chemistry to Aaron Ciechanover, Avram Hershko and Irwin Rose.

The covalent attachment of ubiquitin to a substrate protein in a process known as ubiquitination or ubiquitylation is a highly adaptable, post-translational modification. Ubiquitination controls a diverse range of cellular processes, including cell cycle and division, differentiation and development, DNA repair, transcriptional regulation and signal transduction [6,7,8,9]. The most notable role of ubiquitination is in the regulation of protein stability. Ubiquitination can act as a molecular signal targeting the modified protein to the proteasome, a large multicatalytic protease. This critical regulatory mechanism controls the turnover and concentration of a huge number of intracellular proteins, in addition to the elimination of damaged or misfolded proteins. A ubiquitinated protein is not always destined for destruction, however, and, in the last decade, a number of important non-proteoyltic roles for ubiquitination have been discovered, including regulating the subcellular localization, activity and function of the targeted protein. An exciting area of current research is the interplay between the ubiquitin proteasome system and transcriptional regulation. In addition to controlling steady state levels, proteolysis of transcription factors has been associated with both limiting the transcriptional output and rather surprisingly, promoting activation of certain transcription factors. Additionally important are the numerous non-proteolytic roles of ubiquitination in transcriptional regulation [9], in which ubiquitin can (i) affect the interaction of transcription factors with co-activators [10]; (ii) determine the localisation of transcriptional activators and repressors [11,12]; (iii) regulate transcription factor binding to target sites on chromatin [13]; (iv) determine duration of transcription factor promoter occupancy [14,15]; and (v) modify histones and chromatin reorganisation [16,17].

The traditional paradigm of transcriptional regulation by the ubiquitin proteasome system is the degradation of transcription factors, both soluble and DNA bound. Thus, the UPS controls the abundance of the protein available for activation and also destabilises transcription factor:DNA complexes to prevent uncontrolled activation and gene expression [18]. Unexpectedly, and perhaps somewhat counter-intuitively, the ubiquitination of certain transcription factors is also required for their activity. Salghetti and colleagues first provided a link between these processes when overlapping degron and transactivation domain (TAD) sequences were observed in the transcription factor Myc [19]. It was subsequently revealed that this overlap was not unique to Myc [18,20] and close to 30 transcription factors are reported to contain over lapping degrons and TADs [9]. Multiple models of ubiquitin-meditated activation have been proposed [9,21,22,23,24]. One such model, ’the suicide model’ suggests that DNA binding triggers the ubiquitination of transcription factors which is essential for activation and initial transcription, while simultaneously targeting the protein for degradation [22,23,25,26].

Ubiquitination is a major point of control of many transcription factors, one of which is nuclear factor (NF)-κB, widely considered to be the master regulator of the immune response. Ubiquitination plays an extensive role in the upstream activation of both the canonical and non-canonical NF-κB pathways, however it is now emerging that post-translational modifications including ubiquitination are critical in regulating the NF-κB transcriptional response [27,28,29]. The role of ubiquitination in cytoplasmic events leading to NF-κB activation has been comprehensively reviewed elsewhere [12,30,31,32,33]. This review will focus on the ubiquitination of individual NF-κB subunits, highlighting the importance of this modification in controlling NF-κB transcriptional activity.

## 2. NF-κB

Often described as a single entity, NF-κB is in fact a family of dimeric transcription factors consisting of five proteins; p65 also known as RelA, RelB, c-Rel, p50 and p52. NF-κB members are characterised by the presence of a conserved N-terminal REL homology domain (RHD), a 300 amino acid motif responsible for DNA-binding, dimerisation and nuclear localisation. NF-κB proteins associate to form homo- and heterodimers, the archetype of which is the p65:p50 heterodimer found in most cell types. p50 and p52 are distinctive family members in that they are generated from proteasomal processing of larger precursor proteins p105 and p100, respectively and do not contain a transactivation domain required to initiate transcription and as such are generally considered to be repressors of NF-κB dependent transcription. NF-κB regulates the inducible expression of over 500 genes (nf-kb.org) [34], the majority of which are critical to immune and inflammatory responses. NF-κB target genes are not limited to immune responses, however, and NF-κB also plays a critical role in normal developmental processes, cellular growth and apoptosis. With such wide ranging targets, is not surprising that aberrant or deregulated NF-κB is associated with numerous pathological states including but not limited to autoimmunity, cancer, neurodegeneration and cardiovascular diseases [35,36].

Typically, in resting cells, NF-κB is maintained in an inactive state through association with of an inhibitor of NF-κB (IκB) protein, the prototypical of which is IκBα [37,38,39]. The IκBs are a family of structurally-related proteins, defined by the presence of multiple copies of ankyrin (ANK) repeats which form a central ankyrin repeat domain (ARD). Although NF-κB:IκBα complexes can shuttle between the cytoplasm and nucleus [40,41,42,43], dominant IκBα and p65 nuclear export sequences ensure the steady state location of these complexes is in the cytoplasm [42,44]. A plethora of stimuli induce NF-κB activation, which requires the IκB kinase (IKK) complex-dependent phosphorylation of IκBα [45,46,47]. Phosphorylation of IκBα at serines 32 and 36 creates a destruction motif recognised by the ubiquitin ligase complex, Skp1–cullin1–F-box protein, beta- transducin repeat-containing proteins (SCF^β−TrCP^) [48,49]. Once polyubiquitinated by SCF^β−TrCP^ [50,51,52,53,54], IκBα is targeted for proteasomeal degradation, thus liberating NF-κB dimers. Retention of NF-κB in the cytoplasm offers a highly sensitive system for responding to environmental stimuli. Inducible degradation of IκBs allows latent NF-κB to rapidly translocate to the nucleus, bind κB sites in the promoter and regulatory regions of NF-κB responsive genes and activate transcription. NF-κB directly activates *Nfkbia* gene expression, encoding IκBα, thereby providing an intrinsic negative feedback mechanism to limit the response. Once resynthesised, IκBa enters the nucleus where it dissociates DNA- bound NF-κB and shuttles it back to the cytoplasm.

Although prompt activation of NF-κB is fundamental in mounting an effective immune response, strict control of NF-κB activity is vital in order to limit the expression of potentially damaging genes and to prevent damage to the host from excessive or prolonged immune activation. While IκBα provides a robust mechanism to inhibit NF-κB, the termination of transcriptional activity, occurs even in the absence of IκBα. Studies by Saccani *et al.* have revealed that regulation of NF-κB is more complex than previously thought and in addition to the classical view of global repression mediated by IκB, NF-κB-induced transcription is selectively controlled in a gene specific manner [15]. Distinct mechanisms coordinate to ensure that the activation and termination of the NF-κB response does not produce an all-or-nothing effect. As mentioned above, activation of the NF-κB pathway relies on post-translational modification of the upstream components, such as the IκBs and the IKK complex. However, it is now apparent that the modification of individual NF-κB subunits is vital to producing signal specific responses [27]. NF-κB subunits are subjected to multiple forms of post-translational modification, including phosphorylation, nitrosylation, acetylation and ubiquitination which can have a major functional impact on the protein. Stimulus-dependent induction of these modifications can affect the ability of NF-κB dimers to bind DNA, interact with IκB proteins, recruit essential co-activators and alter the stability of the protein. The barcode hypothesis suggests that these modifications can act alone or in combination to generate distinct patterns that function to direct transcription in a gene-specific fashion [28,55].

### Degradation of NF-κB

Much of our understanding on the role of ubiquitination in regulating NF-κB stems from studies on p65 and, at present, information on other NF-κB subunits is relatively limited. In addition to regulating steady state levels of p65, ubiquitination p65 is required for efficient termination of NF-κB transcription. NF-κB chromatin interactions are highly dynamic and the turnover of promoter bound p65 is reported to occur in less than 30 seconds [56]. Such transient promoter occupancy likely prevents sustained transcription from a single NF-κB complex and may allow rapid exchange of NF-κB dimers, a mechanism that has been suggested to allow temporal fine-tuning of the NF-κB response [56,57]. Furthermore, it allows promoters to continuously sample the nucleoplasm and receive input from any regulatory mechanism. While some NF-κB dimers can selectively bind NF-κB target promoters, considerable redundancy exists and many genes are regulated by more than one dimer [58,59]. However, even after dimer binding, the transcriptional output is not always equivalent. Distinct kinetics, dimer-specific synergy with other transcription factors and interactions with co-factors can regulate discrete gene sets following DNA binding [58,60]. Moreover, as evident from knockout mouse models and cell lines, individual NF-κB subunits also have individual functions [58,61]. Inhibitory p50 homodimers, for example, can compete with p65-containing heterodimers for κB site binding, thereby suppressing NF-κB-dependent transcription. The differential expression of a single NF-κB subunit can also affect the abundance and composition of NF-κB dimers within in the cell. A shift in balance between transcriptionally active and repressive NF-κB dimers can have profound consequences on the transcriptional response. Considerable work in recent years has identified a number of regulators of NF-κB degradation and while many gaps in our knowledge still exist, we can begin to elucidate the mechanisms involved. The key components and basic principles of NF-κB ubiquitination will be discussed in later sections, but first, an introduction to the ubiquitin proteasome system will be given.

## 3. Ubiquitin Proteasome System

### 3.1. Ubiquitin Cascade

**Figure 1:**
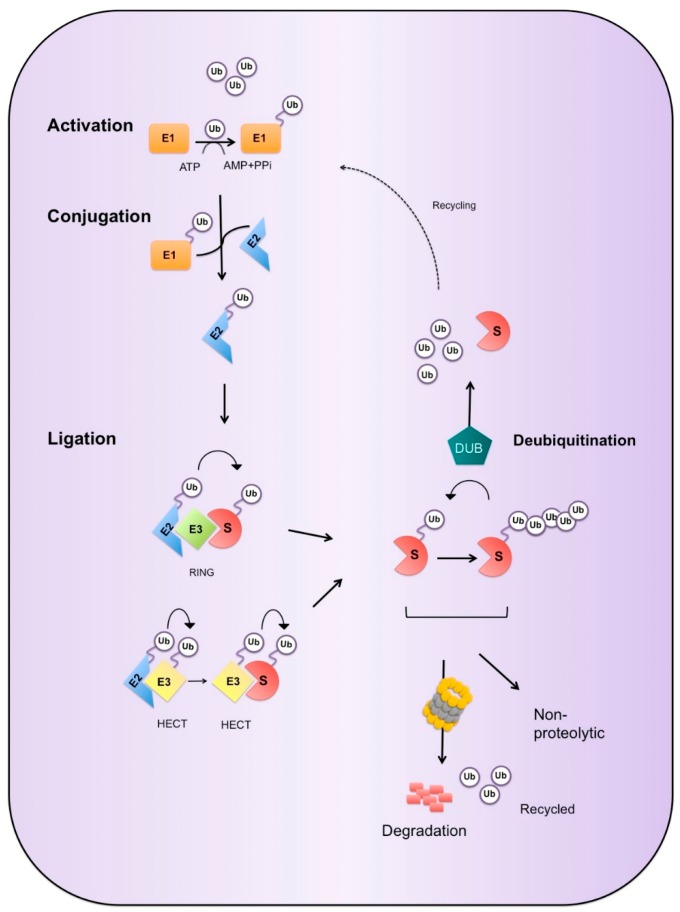
Ubiquitination Cascade. Ubiquitination of a substrate protein (S) occurs via a sequential enzymatic reaction. The initial step involves the ATP-dependent activation of ubiquitin (Ub) by the ubiquitin-activating enzyme (E1) through adenylation of the C-terminal carboxyl group. Once activated, ubiquitin is transferred via a thiolester linkage, to the active site cysteine residue on the E1 with the release of pyrophosphates (PPi) and adenosine monophosphate (AMP). Ubiquitin is next transferred a sulfhydryl group on the ubiquitin-conjugating enzymes (E2) via a transthioesterification reaction. In the final step, a ubiquitin ligase (E3) catalyzes the transfer of ubiquitin to the substrate protein. The two main classes of E3 enzymes are depicted here, HECT (Homologous to the E6-AP C-Terminal domain) and RING (Really Interesting New Gene). Depending on the E3, ubiquitination can occur by direct transfer to the substrate from the E2 or after thioester formation of ubiquitin with the E3. The outcome of the ubiquitinated protein is dependent on type of ubiquitin signal and can involve both proteolytic and non-proteolytic fates. Ubiquitination is also reversible, deubiquiting enzymes (DUB) can hydrolyse the isopeptide bond between ubiquitin and the targeted substrate or the peptide bond between individual ubiquitins to facilitate complete removal or modification of the ubiquitin signal. DUBs are also present in the proteasome, which cleave ubiquitin from its substrate, ubiquitin is then recycled back into the ubiquitin system to maintain a pool of free ubiquitin.

Mammalian ubiquitin is encoded by four genes, UBA52 and RPS27a, which each code for a single copy of ubiquitin [62,63], and UbB and UbC that produce head to tail repeats of ubiquitin, typically four and nine repeats, respectively [62], which are cleaved to produce free ubiquitin by deubiquitnase enzymes [64]. Covalent attachment of a ubiquitin molecule to a target protein is achieved by a three component enzymatic system composed of a ubiquitin-activating enzymes (E1), a ubiquitin-conjugating enzyme (E2) and a ubiquitin ligase (E3) [65] (Figure 1). The first step of this sequential process requires the ATP-dependent activation of ubiquitin, in which the C-terminal carboxyl group of ubiquitin becomes linked to the sulfhydryl group of an E1 via a high-energy thioester bond. Activated ubiquitin is next transferred to the active site cysteine of the E2 ubiquitin-conjugating enzyme. The E2 then acts with the E3 ubiquitin ligase to conjugate ubiquitin to the substrate protein via an isopeptide bond between the C-terminal glycine of ubiquitin and the substrate protein typically but not exclusively at lysine residue [66]. In some cases, ubiquitin can be conjugated to non-lysine residues, such as serine, threonine and cysteine, in addition to the α-amino group of the substrate’s N-terminal residue. The C-terminal glycine of ubiquitin is attached to these non-conventional sites via an ester bond between a serine or threonine on the substrate whereas a thiolester bond is created between a cysteine and the C-terminal glycine [67].

The ubiquitination process may be terminated after the attachment of a single ubiquitin moiety (mono–ubiquitination) or repeated either with additional ubiquitin molecules linked to the ubiquitin molecule previously attached to the substrate, forming a polyubiquitin chain or to another lysine residue of the substrate protein yielding a multi-mono-ubiquitinated protein. To date, only two E1s, UBA1 and UBA5, have been identified in humans, whereas there is an estimated 50 E2s and >600 E3s [68], the combinations of which confer great specificity to the cascade [69]. Typically, a single E2 can interact with multiple E3s, which in turn selectively bind many substrate proteins acting as the substrate recognition molecule. The hierarchical and stepwise nature of the reaction ensures a tightly regulated mechanism for specifically targeting thousands of proteins through a single cascade.

### 3.2. Enzymes of the UPS

#### 3.2.1. E3 Ligases

The E3 ligases represent the largest and diverse category of enzymes in the ubiquitination cascade and can be characterised into three distinct classes: Really interesting new gene (RING) and RING-like (consisting of plant homeodomain/leukemia-associated protein (PHD/LAP) and U-box); homology to E6AP C terminus (HECT); and the RING-between-RING (RBR) [68,70,71,72]. Each ligase family contains conserved structural domains and the mechanism by which ubiquitin is transferred from the E2 to the substrate occurs by two general mechanisms. RING-type ligases bind the E2 and the substrate simultaneously to catalyse the transfer of ubiquitin directly from the E2 to the substrate. In contrast, HECT and RBR ligases are involved in a two-step reaction in which the E3 forms an E3-ubiquitin intermediate. Ubiquitin is first transferred from the E2 to the active site cysteine of the E3 via trans-thiolation before conjugation to a substrate. In addition to functioning as monomers, RING-type ligases may also form homo- and heterodimers and can also exist as part of large multi-subunit assemblies, the most notable of which is the Cullin-RING ligase (CRL) superfamily. CRL families are characterised by the presence of a cullin scaffold protein, (CUL-1, 2, 3, 4a, 4b, 5, or 7, 9/PARC), a small RING protein, typically Rbx1, Roc or Hrt1, and various adaptors, which recruit substrates to the ligase complex.

#### 3.2.2. Deubiquitinases

While the outcome of ubiquitination is largely determined by the nature of the ubiquitin chain, modification by ubiquitin does not commit a protein to a particular fate. Similar to other post-translational modifications, ubiquitination is reversible and a superfamily of highly specific proteases called deubiquitinating enzymes, deubiquitinases (DUBs) mediate the removal of ubiquitin from substrate proteins [73,74,75]. Opposing the ubiquitination process, DUBs hydrolyse the isopeptide bond between the ubiquitin and substrate protein. Deubiquitinases, thereby, counterbalance E3 ligase activity and by removing or modifying a potentially degradative signal can rescue a protein from proteolysis. In addition to complete removal of a polyubiquitin chain from a substrate, some deubiquitinases can cleave the isopeptide or peptide bonds in between or at the distal end of a polyubiquitin chain [76]. Trimming the polyubiquitin chain in this manner serves to edit the signal either by modifying the chain length or by facilitating the exchange of one type of ubiquitin linkage for another. Deubiquitination can also modify non-degradative signals in turn regulating signalling cascades that are triggered by these non-proteolytic ubiquitin chains. There are approximately 100 DUBs encoded in the human genome, which can be divided into five different families according to their protease domain [75,77]. The majority of DUBs are cysteine proteases and include the ubiquitin carboxy-terminal hydrolases (UCHs), ubiquitin specific proteases (USPs), ovarian tumor proteases (OTUs) and Machado-Joseph diseases proteases (Josephins) families, but there is also a small group of zinc metalloproteases referred to as JAB1/MPN/Mov34 (JAMMs). To date, two members of the USP family of DUBS have been found to deubiquitinate NF-κB subunits, USP7 [78] and USP48 [79,80].

### 3.3. Ubiquitin Mediated Degradation

Ubiquitin contains seven lysine residues (Lys6, Lys11, Lys27, Lys29, Lys33, Lys48 and Lys63), which are utilized during the formation of a polyubiquitin chain. Ubiquitin monomers are linked via an isopeptide bond between one of these internal lysine residues and the C-terminal glycine of the ubiquitin previously ligated to the substrate. In addition, ubiquitin can be covalently attached via a peptide bond to a methionine of another ubiquitin in a ‘head to tail’ manner which is known as linear or Met1-linked ubiquitination [81].K48 and K63 linked ubiquitin linkages were the first types of polyubiquitin chains to be identified [82] and extensive studies have defined essential roles for polyubiquitin chains of these types in protein degradation and cellular signalling respectively [67]. While mass spectrometry has identified all types of ubiquitin linkages in mammalian cells, comparatively little is known about the roles of the remaining atypical chains but they may involve both degradative and non-degradative functions [83,84]. Although it was once assumed polyubiquitin chains were exclusively homogenous in nature, heterogeneous or mixed polyubiquitin chains, which contain different ubiquitin–ubiquitin linkages and forked chains in which multiple ubiquitin molecules are conjugated to the previously attached ubiquitin have also been described [66,84]. The ability of ubiquitin to form a diverse array of linkages with chains of varying length produce a multitude of signals that can be recognised and decoded by proteins containing a specialised ubiquitin binding domain. Polyubiquitin chains assembled through lysine 48 are probably the best characterised type of linkage and are widely recognised as the canonical signal for degradation by the 26S proteasome, a 2.5-MDa molecular machine responsible for proteolysis. The minimum signal required for efficient targeting to the proteasome was initially accepted to be K48 linked tetra-ubiquitin [85], however recent studies have shown that modification by a single ubiquitin is sufficient for recognition by the proteasome [86,87,88]. Furthermore, recent work has proposed that in addition to K48 linkages, K11 linkages can act as a second type of degradation signal, forming homogenous and K48 branched chains which has been found to enhance the degradation of some substrates [89,90].

## 4. Ubiquitination of NF-κB

**Figure 2:**
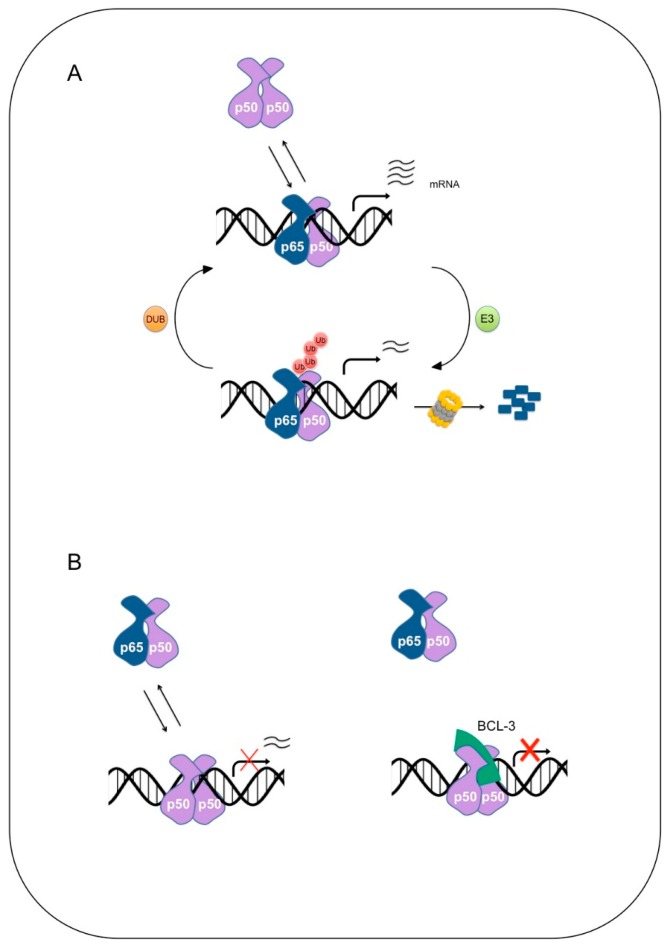
Regulation of NF-κB dependent transcription by ubiquitination. (**A**) Once liberated from cytoplasmic retention, NF-κB dimers are free to translocate to the nucleus and bind κB sites in the promoter and regulatory regions of NF-κB responsive genes to activate transcription. Promoter occupancy is highly dynamic and NF-κB dimers are in constant competition for binding at these sites. DNA binding triggers the ubiquitination and subsequent degradation of promoter-bound NF-κB allowing the promoter to be reloaded by another NF-κB dimer. Deubiquitases (DUB) oppose the action of the E3 ubiquitin ligase, removing the degradative signal and in turn extending the occupancy time of the dimer; (**B**) many NF-κB dimers can bind to the same κB sites. As p50 does not contain a transactivation domain, binding of p50 homodimers to κB sites represses the transcription of NF-κB dependent genes. As with p65, DNA binding triggers the ubiquitination of p50 however this can be inhibited by the nuclear IκB protein, BCL-3, extending the occupancy time of inhibitory p50 homodimers.

Although almost all NF-κB subunits have been shown to be ubiquitinated, the focus in recent years has largely remained on p65. Many regulators of NF-κB ubiquitination have been identified including E3 ligases and deubiquitinases however a disproportional number of these are related to p65 and our current understanding of the ubiquitination of other subunits is limited in comparison. Ubiquitin-mediated degradation of p65 is induced by multiple TLR and TNFR ligands [15,91]. However, in contrast to IκBα, not all p65 protein is degraded following activation of the NF-κB pathway. Ubiquitination of p65 occurs predominantly in the nucleus, and inhibition of proteasome activity appears to selectively stabilise nuclear p65 with minimal effect on the cytoplasmic fraction [15,92]. Fitting with this, binding to DNA is a critical trigger of p65 ubiquitination, thus a DNA binding defective p65 mutant is resistant to ubiquitination [15]. The requirement for DNA binding ensures only a specific fraction of nuclear p65 is ubiquitinated and targeted for degradation, removing it from DNA (Figure 2). Many components of the UPS are recruited to NF-κB target gene promoters including Sug1, a 19S proteasome ATPase, and multiple ubiquitin ligases [15,93,94]. Moreover, ubiquitinated p65 has also been found associated with chromatin suggesting that these promoter regions may also represent sites of p65 degradation [95]. In the absence of p65 degradation, achieved either through proteasome inhibition or through the expression of an ubiquitination resistant version of p65, promoter occupancy of p65 is dramatically extended, resulting in sustained expression of NF-κB-dependent genes [15,92]. Interestingly, inhibition of proteasome activity does not impair dissociation of p65 from all NF-κB target promoters, suggesting that ubiquitination may only be critical in limiting the expression of specific NF-κB target genes [15]. Alternatively, the role of ubiquitination at some promoters may be to remove NF-κB form DNA independently of subsequent proteasomal degradation.

It is important to note that in addition to degradative K48 linked polyubiquitination, p65 ubiquitination has been shown to involve a number of other ubiquitin linkages including K29, K33 and K63, however the functional consequences of these non-degradative forms of ubiquitinated p65 is currently unknown [95]. p65 has also been reported to be regulated by mono-ubiquitination which does not promote p65 degradation. The mono-ubiquitination of p65 is thought to promote nuclear retention of p65 and may inhibit p65 transcriptional activity by inhibiting interaction with the transcription co activator CBP [96].

Mass spectrometry analysis has identified numerous lysine acceptor sites on p65. Two independent studies reported ubiquitinated p65 at K62, K123 and K315 following proteasomal inhibition [95,96], however one of these studies also reported the ubiquitination of p65 at K56, K79, K195, and K310 [95]. It has been suggested that ubiquitination of p65 is highly promiscuous as mutation of virtually all lysines is required to completely abolish p65 ubiquitination [95]. However, it is currently unknown if a single p65 molecule is ubiquitinated on multiple sites simultaneously or if modification at an individual lysine residue occurs in isolation. The presence of many ubiquitin acceptor sites may be due to the binding specificities of different E3 ligases. Indeed several E3 ligases of p65 have been described and while the ubiquitination sites for all ligases have not yet been identified, the sites of those that have, do not appear to overlap. Why multiple p65 ligases appear to be involved in the regulation of NF-κB transcriptional activity is also unknown and while some NF-κB target genes, such as *Il6*, are regulated by more than one ligase, the available data demonstrates selective regulation of other target genes. It is possible that these ligases bind specific p65 containing dimers or have predominant roles depending on the cell-type or stimulus.

**Figure 3:**
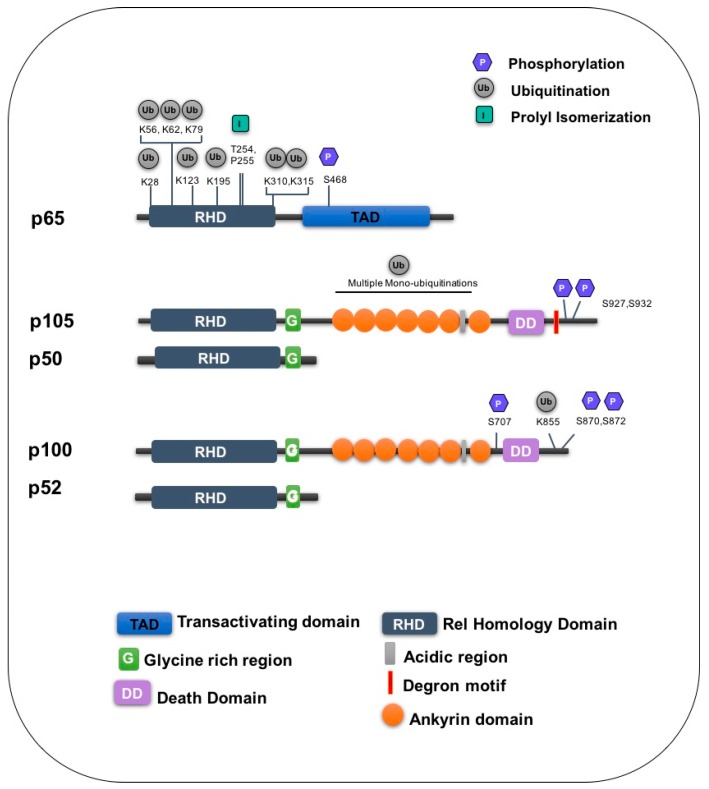
Post-translational modifications associated with the ubiquitination of NF-κB subunits. Schematic representation of the NF-κB subunits with known post-translational modifications associated with NF-κB ubiquitination indicated. RHD: REL homology domain, TAD: transactivating domain, G:glycine rich region, DD: Death domain.

Interestingly, a number of the ubiquitin acceptor sites identified by these mass spectrometry studies were found to overlap with known acetylation sites on p65. Consistent with overlapping lysine residues, these modifications were found to impact each other. Increased p65 ubiquitination results in decreased p65 actelylation, while conversely promoting acetylation through expression of CREB-binding protein, a histone acetyltransferase inhibits the ubiquitination of p65 [95]. Whether the interplay between acetylation and ubiquitination is solely due to competition for lysine sites or if other mechanisms are involved is presently unclear. Modification of these residues can have diverse consequences on NF-κB dependent transcription and depending on the target gene can result in both the increased or repressed gene expression [95]. p65 is also subject to methylation at lysine residues modified by both acetylation and ubiquitination suggesting that cross-talk between several post-translational modifications may be important in regulating p65 and controlling transcription in a gene-specific manner [97,98,99,100]. Indeed, a number of NF-κB post-translation modifications associated with ubiquitination have been identified and are summarised in Figure 3 and Table 1.

Gene-specific regulation of transcriptional activity has been demonstrated for other post-translation modifications of NF-κB subunits [28,29,97,101]. For example, phosphorylation of p65 at serine 468 has been shown to both positively and negatively regulate TNFα-induced NF-κB target gene expression [28,102]. Differential regulation of gene expression by p65 phosphorylation may also be mediated through ubiquitination. Phosphorylation of serine 468 of p65 for example promotes its ubiquitination and proteasomal degradation while mutation of this residue impairs ubiquitination resulting in an extended p65 half-life and increased occupancy of p65 on the promoters of specific genes [28,102]. Phosphorylation at this site promotes p65 binding to COMMD1, cullin2 and GCN5, components of a multimeric ligase complex mediating p65 ubiquitination [102,103]. p65 phosphorylated at 468 can be found associated with the promoters of selective NF-κB genes and is thought to contribute to the termination NF-κB gene expression through the removal of chromatin bound p65 [102]. p65 is phosphorylated on a number of other sites, many of which regulate p65 stability and function and while outside the scope of this review are discussed in detail elsewhere in this series [104].

Similar to p65, the ubiquitination of p50 is induced in response to TLR and TNFR activation, however, unlike its more studied counterpart, little is known about the components or the mechanisms involved. As reported for p65, the ubiquitination of a mutant of p50 that is defective in DNA binding is substantially reduced, suggesting that DNA binding also acts as a trigger for p50 ubiquitination. This is supported by the fact that significant ubiquitination of p50 is observed only after stimulation, when p50 is free to translocate to the nucleus. The E3 ligase/s required for p50 ubiquitination have yet to be identified and the available data on p50 ubiquitination stems from studies on the atypical IκB protein, BCL-3. BCL-3 selectively interacts with homodimers of p50 and p52 and is characterized as a member of the IκB family due to the presence of seven ankyrin repeats [105,106,107]. It is considered atypical however, as, unlike classical IκBs, BCL-3 resides predominantly in the nucleus and is not degraded upon IKK activation. *Bcl3* was first identified as a putative proto-oncogene [108] and while overexpression has been associated with some cancer types [109], BCL-3 also has an critical role in limiting NF-κB transcriptional activity in immune cells and promoting endotoxin tolerance to LPS [110,111].

In the absence of BCL-3, p50 is hyper-ubiquitinated resulting in a substantial reduction in the half-life of p50 in macrophages [110]. Without stable p50 homodimers, binding of p65 containing dimers to the promoters of NF-κB target genes such as *Tnf* and *Cxcl2* is increased both in resting and activated *Bcl3^−/−^* cells leading to increased gene expression (Figure 2). As a result, *Bcl3*^−/−^ cells and mice are hypersensitive to TLR activation and LPS-induced septic shock. BCL-3 promotes stable inhibitory p50 homodimer:DNA complexes through the inhibition of Lys-48 linked ubiquitination and degradation of p50. How BCL-3 inhibits p50 ubiquitination is currently unknown, however interaction with p50 has been shown to be essential for the anti-inflammatory properties of BCL-3 [112]. Cells that express a mutant of p50 that can no longer interact with BCL-3 are hyper responsive to TNFα stimulation and demonstrate increased p50 ubiquitination and turnover.

Unlike p65 and p50, DNA binding is not essential for RelB ubiquitination and may consist of linkages other than Lys-48 and Lys-63. Ubiquitination of RelB is reported to enhance its transcriptional activity suggesting that ubiquitination may serve non-proteolytic roles for this subunit, however, it is possible that RelB is modified with multiple types of polyubiquitin chains with distinct functions. Inducible degradation of RelB has been reported in primary T cells and a number of both B- and T-cell cell lines, however, while stimulation also induces ubiquitination the it is unclear if this is linked to ubiquitination [113,114,115]. Degradation of RelB appears to be signal specific however, as stimulation of lymphocytes with phorbol myristate acetate (PMA)/imonoymcin and anti-CD3/CD28, but not TNF induces phosphorylation dependent proteasomal degradation [114]. The degradation of RelB is a multistep process, occurring after N-terminal cleavage by the paracaspase Malt1 at Arg 85 [113,114].

## 5. E3 Ligases of NF-κB

### 5.1. SOCS1

Suppressor of cytokine signalling 1 (SOCS1) was the first NF-κB E3 ligase to be identified. SOCS1 is a member of the CIS–SOCS (SOCS1-7 and CIS) family of intracellular proteins which regulate cytokine responses of a number of immune cells [127]. SOCS proteins contain variable a N-terminal domain and two conserved motifs; a central SH2 domain and a C-terminal SOCS box. The SOCS box is a ~40 amino acid domain not exclusive to SOCS proteins that acts as the substrate recognition component of ECS ubiquitin ligase complexes. SOCS1 binds to and induces the ubiquitination and degradation of p65 as part of a multisubunit complex containing, elongin c, cullin2 and Rbx1 designated ECS^(SOCS1)^ [119]. Copper metabolism Murr1 domain-containing 1 (COMMD1) interacts with this ECS^(SOCS1)^ complex and stabilises the interaction between SOCS1 and p65, promoting p65 ubiquitination [128]. The ubiquitination of p65 is greatly impaired in COMMD1^−/−^ myeloid cells resulting in enhanced expression of certain NF-κB target genes [129]. COMMD1 appears to selectively regulate a subset of LPS-induced target genes, as while expression of *Mx1* and *Cd86* for example are increased in the absence of COMMD1, no affect is seen on *Il6* and *Tnfaip3* expression.

As SOCS1 and COMMD1 are predominantly localised to the cytoplasm, some debate exists over whether the ECS^(SOCS1)^ complex can regulate the ubiquitination of nuclear p65 [91,130,131]. In recent years however, nuclear-cytoplasmic transport of both SOCS1 and COMMD1 have been reported [132,133], in addition SOCS1 interaction with nuclear p65 has also been shown [134]. Furthermore, COMMD1 promotes the ubiquitination and proteasomal degradation of p65 in the nucleus and is recruited to NF-κB target promoters where it decreases the duration of p65 chromatin interactions to inhibit NF-κB mediated transcription [93,129]. The histone acetyltransferase GCN5 acts as a co factor for the ECS^(SOCS1)^ ligase complex and promotes p65 ubiquitination and acts independent of its enzymatic activity [103]. GCN5 binds to both COMMD1 and p65 acting as an accessory factor for the E3 ligase complex. Similar to COMMD1 [93], GCN5 is inducibly recruited to the promoters of NF-κB target genes and contributes to the regulation of chromatin-associated p65. Deletion of GCN5 results in increased p65 in the nucleus and increased promoter occupancy.

### 5.2. ORF73

An additional ECS type ligase complex composed of a viral SOCS box protein, ORF73 (ECS^(ORF73)^), has also been shown to promote the ubiquitination and degradation of nuclear p65, in turn inhibiting host NF-κB transcriptional activity [135]. ORF73 encoded by murid herpesvirus-4(MuHV-4), a murine gammaherpesvirus related to human viruses, such as Epstein-Barr virus and Kaposi's sarcoma-associated herpesvirus, interacts with ElonginC and Cullin5 via its SOCS box motif to mediate p65 ubiquitination. Disruption of the ORF73 SOCS box in MuHV-4 suppresses viral expansion in germinal centre B cells and prevents persistent infection in mice. ORF73 is thought to mimic the host ECS^(SOCS1)^ complex inhibiting TNF induced NF-κB activation which, may contribute to immune evasion by (MuHV-4) and to expansion of the host pool of latently infected cells required for persistence viral infection.

### 5.3. PDLIM2

The PDZ-LIM domain protein, PDLIM2 (also known as SLIM [136] and mystique [137]) is a member of the actinin-associated LIM protein (ALP) subfamily [138]. Depending on the cell type and differentiation state PDLIM2 may be localised to nucleus or cytoplasm and can associate with the cytoskeletal [91,136,137,138,139,140]. In lymphocytes PDLIM2 has been shown to regulate the nuclear stability of several proteins including p65, STAT and TAX whereas in epithelial cells, PDLIM2 is required for adhesion and cellular migration. PDLIM2 was first identified an E3 ligase regulating STAT-mediated signal transduction [136]. PDLIM2 binds to STAT 4 and promotes its ubiquitination and degradation to inhibit STAT-induced gene expression. PDLIM2 contains a single LIM domain which is a protein -protein interaction motif consisting of a cysteine rich zinc finger domain similar to the RING domain found in many E3 ligases. Comparable to its role in STAT regulation, PDLIM2 binds to and promotes the ubiquitination of p65, however, the ability to ubiquitinate p65 *in vitro* has not yet been demonstrated [91]. In the absence of PDLIM2, the ubiquitination of p65 following TLR activation is greatly impaired resulting in accumulation of p65 in the nucleus and enhanced NF-κB activity of dendritic cells. As a consequence, *Pdlim2^−/−^* mice exhibit increased mortality to systemic administration of LPS compared to wild type mice. Independent of its LIM domain, PDLIM2 also promotes intranuclear trafficking of p65 from soluble to insoluble nuclear compartments, targeting p65 to PML nuclear bodies via its PDZ domain. Recently, the chaperone protein heat shock protein of 70 kD (HSP70) was found to be required for the proteasome-dependent degradation of p65 in this insoluble nuclear fraction [141]. It is thought that together with BCL2-associated athanogene 1 (BAG-1), HSP70 promotes degradation of p65 by PDLIM2 by promoting transport of the NF-κB-PDLIM2 complex to the proteasome. Nucleolar translocation of p65 has previously been shown to repress NF-κB dependent transcription and inhibit stress-induced apoptosis of SW480 human colon adenocarcinoma cells [142]. Under these conditions ubiquitination of p65 precedes nucleolar translocation and has been suggested to act as signal to target p65 to the nucleolus [142,143]. Redistribution of p65 from the nucleoplasm to these insoluble nuclear compartments and the nucleolus is thought to physically sequester p65 from target promoters acting as an alternative mechanism to limit NF-κB transcriptional activity [91,142,143].

### 5.4. PPARγ

Peroxisome proliferator activated receptor (PPAR)-γ is a member of the superfamily of nuclear hormone factors. In addition to PPARγ, there are two other PPAR subtypes PPARα and PPAR β/δ, which are important in regulating energy homoeostasis, lipid biosynthesis and glucose metabolism. While PPARγ expression was initially thought to be restricted to adipocytes, it is now clear that PPARγ is expressed in multiple cell types [144]. In addition to its role in metabolism, PPARγ also been implicated in negatively regulating the immune response and the expression of inflammatory cytokines in macrophage. Several studies have reported that PPARγ binds to p65 and inhibits NF-κB transcriptional activity *in vitro* however the mechanism of action remained unclear until recently [145,146,147]. PPARγ contains a RING finger domain and independently of its transcriptional activity, it has been revealed that PPARγ acts as an E3 ligase to promote the ubiquitination and degradation of p65 [116]. Overexpression of PPARγ induces K48 linked ubiquitination of p65 resulting in a rapid decrease in p65 half-life where as p65 stability is substantially increased in PPARγ^−/−^ MEFs. PPARγ interacts with p65 in MEFs under basal conditions however binding and degradation is enhanced following TNF and LPS stimulation. Mass spectroscopy analysis identified lysine 28 of p65 as the critical polyubiquitin target site of PPARγ and a K28R p65 mutant is resistant to PPARγ mediated ubiquitination and degradation.

### 5.5. ING4

Inhibitor of growth (ING) 4 is a member of the ING family of chromatin-modifying proteins, which includes ING1-5. ING proteins have been implicated in a number of essential cellular events including DNA repair, tumour angiogenesis cell growth and proliferation and are classified as type II tumour suppressors. Dysregulated ING4 expression has been associated multiple cancers including gastric, ovarian, colon, brain and breast cancers in addition to melanomas and hepatocellular carcinomas. ING4 interaction with p65 was first identified in human U87MG glioblastoma cells, which have constitutively activated NF-κB [148]. Subsequently, overexpression and knock down experiments in glioma cell lines suggested that ING4 may regulate NF-κB activity by binding to promoter bound p65 [94]. ING4 is inducibly recruited to the promoters of the NF-κB-regulated genes, *Cox2* and *Mmp* following Phorbol 12-myristate 13-acetate (PMA) and TNFα. Binding of ING4 at the *Cox2* promoter corresponded with a reduction of p65/p300 bound complexes and an increase in p65/HDAC1 bound complexes. p65 promoter occupancy is increased in ING4^−/−^ macrophages resulting in increased expression of the LPS-induced NF-KB target genes *Il6*, *Ip10* and *Ifnb* [149]. Interestingly, ING4 also negatively regulates only a subset of NF-κB target genes and appears to be required for maximal expression *Tnf* following LPS.

Together with the E2 enzyme UbcH3, ING4 was recently found to promote the K48 linked ubiquitination and proteasome-dependent degradation of p65 via its plant homeodomain (PHD) motif [117]. The PHD domain is a RING like zinc finger protein that shares structural similarity with LIM and RING domains. Silencing of ING4 in 293T cells increases the half-life and TNFα-induced nuclear accumulation of p65. Lysine 62 of p65 was found to be critical for ING4 mediated ubiquitination and a K62R p65 mutant is resistant to ING4 induced ubiquitination and degradation.

### 5.6. HSV-1 ICP0

ICP0 is an immediate-early gene product of herpes simplex virus 1 which has essential roles in regulating both lytic and latent infections [150]. ICP0 interacts with a number of cellular proteins however recently ICP0 has been shown to interact with p65 and p50 NF-κB subunits to inhibit NF-κB mediated proinflammatory cytokine production [151]. ICP0 contains a RING finger domain located at its amino terminus which mediates its E3 ligase activity and induces the proteasome dependent degradation of several cellular proteins [152]. While ICP0 interacts with both p65 and p50, ICP0 only induces the proteasomal dependent degradation of p50 [151]. ICP0 targets p50 for K48-linked ubiquitintaion dependent on its RING domain and inhibits NF-κB activity which may contribute to suppression of the NF-κB mediated antiviral response.

### 5.7. Peli 1

Peli 1 a member of the Peli (Pelino) family of RING ligases has been implicated in promoting the ubiquitination of c-Rel in T-cells [153]. Although ubiquitination of c-Rel was described almost twenty years ago, little was known about this process until relatively recently. Chen and colleagues first reported ubiquitinated c-Rel in serum stimulated Jurkat T-cells [154]. The authors also demonstrated that c-Rel degradation was mediated by the proteasome pathway and suggested that the c-terminal domain of c-Rel was important in facilitating this degradation. c-Rel also undergoes Peli-1 dependent ubiquitination in T-cells stimulated anti CD3 and anti CD28. Peli 1 deficiency results in the nuclear accumulation of c-Rel in activated T-cells while c-Rel ubiquitination is largely inhibited. Peli 1 can promote the formation of K48 and K63 linked ubiquitin chains, however Peli 1 appears to induce only K48 linked ubiquitination of c-Rel. NF-κB activity is increased in *Peli1^−/−^* cells stimulated with both anti CD3/CD28 PMA and ionomycin suggesting that Peli 1 is required to limit c-Rel in activated T-cells. However, while endogenous interaction between Peli 1 and c-Rel has been shown, evidence of direct ubiquitination of c-Rel by Peli 1 is currently lacking.

### 5.8. cIAP

Recent work has also shown that c-Rel undergoes ubiquitination in macrophage which is regulated by an alternative ligase complex involving the adaptor molecule TNF receptor-associated factor (TRAF)-2 and cIAP which is involved in the ubiquitination of NIK [155,156]. TRAF-2 deficient macrophages have elevated steady state and LPS-induced levels of c-Rel compared to wildtype cells whereas the K48 linked ubiquitination of c-Rel is attenuated [157]. Moreover, chromatin immunoprecipitation analysis revealed enhanced DNA-binding activity of c-Rel at the *Il12a* and *Il12b* promoters in the absence of TRAF-2. Accumulation of c-Rel and increased NF-κB gene expression is also seen in cells deficient in TRAF-3, a known binding partner of TRAF-2. In overexpression studies, TRAF-2 was found to strongly associate with c-Rel only in the presence of TRAF-3, while both TRAF-2 and TRAF-3 were required for c-Rel interaction with cIAP [157]. Inhibition of cIAP promoted the LPS-induced expression of a number of cytokines in wildtype cells but had no effect in TRAF-2 knockout macrophages. Taken together these data suggest that a cIAP is recruited to c-Rel via a TRAF-2 and TRAF-3 containing complex to promote the ubiquitination and proteasomal degradation of c-Rel. It is important to note however, that these proteins interact with many other components of the TLR pathway [158,159] and similar to Peli 1, direct ubiquitination of c-Rel by cIAP has not been shown.

### 5.9. HERC3

A recent study has also identified a role for the HERC3 E3 ligase in regulating the ubiquitination and degradation of p65 [160]. HERC3 binds to p65 following liberation from IκBα and its overexpression leads to p65 ubiquitination and degradation. Remarkably this is independent of HERC3 E3 ligase activity but appears dependent on ubiquilin 1, suggesting the involvement of an additional E3 ligase. The exact role of HERC3 is unclear since knockdown of HERC3 alone is not sufficient to significantly increase TNFα-induced NF-κB-dependent gene expression, whereas knockdown of ubiquilin-1 appears to regulate transcription in a gene specific manner.

## 6. Deubiquitinases of NF-κB

### 6.1. USP7

USP7 (HAUSP) was the first DUB identified that removes poly-ubiquitin chains from p65. USP7 is a cysteine protease, localised in the nucleus, and preferentially cleaves substrate-attached monoubiquitin and short chains of all linkages with the exception of linear ubiquitin chains [161,162,163,164,165]. USP7 interacts with the p65, c-Rel, p50, p52, and RelB NF-κB subunits although its deubiquitinase activity has only been demonstrated for p65 [78]. Blockade of USP7 enzymatic activity leads to a dramatic increase in the ubiquitination and proteasomal degradation of p65 and profoundly inhibits NF-κB transcriptional activity. The identification of USP7 as a p65 DUB revealed that NF-κB ubiquitination occurs to an even greater extent than previous studies on the E3 ligases of NF-κB suggested. These studies demonstrate that NF-κB transcriptional activity is controlled by a balance of ubiquitination and deubiquitination. USP7 preferentially interacts with DNA bound p65 and is recruited to NF-κB target gene promoters along with p65, suggesting that deubiquitination of p65 may occur at sites of transcription. The molecular determinants of the recognition of p65 as a substrate by USP7 are not clear, but the phosphorylation of p65 at S536, but not S468, promotes interaction with USP7. The importance of USP7 in promoting NF-κB transcriptional responses highlighted it as a novel therapeutic target for the inhibition of NF-κB activity at the level of gene promoter. However, USP7 deubiquitinates a wide range of different substrates including MDM2/HDM2 [163,166,167,168], p53 [163,166,167,168], PTEN [167], FOXO4 [167], PCNA [169] and Rad18 [170]. Thus, the pharmacological targeting of USP7 peptidase activity is likely to have a significant impact on a number of important cellular pathways in addition to NF-κB.

### 6.2. USP48

USP48 (previously referred to as USP31) is also a member of the USP family of DUBs and demonstrates a higher activity towards K63-linked polyubiquitin chains relative to K48-linked polyubiquitin. The role of USP48 in the regulation of NF-κB was initially identified following a yeast two hybrid screen for TRAF2 interacting proteins [79]. Initial studies found that overexpression of USP48 inhibits NF-κB activity downstream of IKKβ and that USP48 also interacts with p65. However, subsequent studies demonstrated that in fact USP48 promotes NF-κB activity by co-operating with the COP9 signalosome to trim K48 linked polyubiquitin chains from p65 in the nucleus [80]. This ubiquitin chain trimming activity confers stability to nuclear p65 and promotes NF-κB transcriptional activity. The activity of UPS48 on NF-κB is enhanced by casein-kinase 2 mediated phosphorylation of USP48 which occurs following TNFα stimulation. It is possible that USP48 may act to stabilise the nucleoplasmic pool of p65 while USP7 may act on chromatin bound p65 to promote stability and transcriptional activity. However, further studies on the interplay of DUBs on the regulation of NF-κB subunits are required to determine the specific roles of individual DUBs.

## 7. Regulation of NF-κB Precursors

Directing a protein to the proteasome typically results in complete degradation but in rare cases, partial proteolysis by the proteasome can yield biologically active protein fragments [171,172]. This partial proteolysis or processing is responsible for the generation of mature NF-κB subunits p50 and p52 from p105 and p100, respectively [173,174,175,176,177]. In addition to post-translational processing from p105, constitutive generation of p50 by a co-translational mechanism has also been proposed which is also dependent on the proteasome but does not require ubiquitination [178,179]. In addition to their primary role as NF-κB precursors, p100 and p105 also act as IκB proteins by virtue of their ankyrin repeat domain [180,181,182,183]. While classical IκB proteins form 1:1 complexes with NF-κB dimers, NF-κB precursors can form large high-molecular weight complexes due to their unique structure comprising both a RHD and ARD [184]. Ubiquitination of NF-κB precursors therefore serves dual roles, partial proteolysis produces active NF-κB subunits which can homo- or hetero-dimerise with other NF-κB family members and complete degradation liberates sequestered NF-κB dimers from inhibition.

### 7.1. p105

p105 processing to p50 may occur constitutively or inducibily. Both types of processing are dependent on ubiquitination but appear to be regulated by independent mechanisms [174,177,185,186,187]. In addition to processing, signal induced ubiquitination can also target p105 for complete proteasomal degradation [186,187]. Following stimulation p105 is phosphorylated on serines 927 and 932 by IKKβ [120], creating a degron or destruction motif recognised by the SCF^β−TrCP^ ubiquitin ligase complex [49], resulting in complete proteolysis of p105 [188,189,190]. In addition to mediating p105 degradation, IKKβ has also been implicated in processing to p50 however, this occurs independently of β-TrCP [191,192].

The glycine rich region (GRR) of p105 found between the RHD and the ankyrin repeat domain spanning residues 372–394 of mouse p105 is an important determinant of proteasomal processing (Figure 3). p105 is degraded from its C-terminus and this region is thought to act as proteasomal stop signal protecting p50 from degradation [193,194,195,196]. While the significance of the GRR has been known for some time, the exact mechanism of p105 processing remained largely unknown, however recent work by Ciechanover and colleagues has begun to elucidate this process [88,191]. Unlike β-TrCP mediated polyubiquitination of p105, processing to p50 does not require the formation of ubiquitin chains. Instead p105 is mono-ubiquitinated on multiple lysines [88,191], which is sufficient to trigger recognition by the proteasome. KIP1 ubiquitination promoting complex (KPC), a RING type ubiquitin ligase complex composed of KPC1 and KPC2 was identified as the ligase involved in ubiquitinating p105 and mediating both basal and signal induced processing [121]. Interestingly over expression of KPC1 was found to inhibit anchorage independent growth of osteosarcoma and glioblastoma cell lines and tumour growth in a mouse xerograft model. In addition to increased p105 processing in tumours over expressing KPC1, expression of a subset of p50 target genes associated with tumour suppressive signals was also increased while a converse reduction in p65 levels was also observed.

### 7.2. p100

In addition to activation of the classical NF-κB pathway mediated by the IKK complex, a second pathway known as the alternative or non-canonical NF-κB pathway is induced by distinct TNFRSF ligands and regulates specific immunological processes including secondary lymphoid organogenesis and B-cell maturation and survival [197,198]. Instead of IκBα degradation, the non-canonical NF-κB pathway relies on the inducible processing of p100 and is characterised by nuclear translocation of the RelB/p52 heterodimer [199]. In contrast to constitutive p105 processing, processing of p100 is signal induced [175] and is dependent on the NIK and IKKα- induced phosphorylation of p100 [123,124]. Signal induced phosphorylation of serines 866 and 870 create a binding site for β-TrCP, which resembles the phosphorylation sites on IκBα required for degradation [200]. As with IκBα degradation, p100 processing is dependent on the SCF^β−TrCP^ ubiquitin ligase complex and lysine 856 of p100 serves as the acceptor site for ubiquitin [122,201].The events succeeding ubiquitination and how p100 is targeted for limited proteolysis are poorly understood. S9 a protein located in the lid of the 19S regulatory particle of the proteasome was identified as an interaction partner of p100 through a yeast two-hybrid study, however, the role of this interaction is currently unclear [202].

p100 also interacts with additional ubiquitin ligase complex comprised of Skp1, Rbx1, Cullin 1 and F-box and WD repeat domain-containing (Fbw7α) [125,126]. Unlike the signal induced processing induced by β-TrCP, constitutive ubiquitination by Fbw7α promotes the proteasomal degradation of p100. Fbw7α mediated ubiquitination is also dependent on phosphorylation, however this requires GSK3β mediated phosphorylation of serine 707 [125,126]. Degradation of p100 is required for efficient activation of the non-canonical pathway and in cells lacking Fbw7α, LTβR-induced NF-κB target gene expression is attenuated [125]. Moreover, the expression of a S707A p100 mutant that is resistant to Fbw7α mediated ubiquitination was found to decrease LTβR–induced association of RelB to the promoters of NF-κB target genes thus inhibiting transcription.

## 8. Concluding Remarks

NF-κB regulates hundreds of genes critical for the inflammatory response which, because of the potential for severe damage to the host from aberrant or unchecked activity, must be strictly controlled. Post-translational modification of NF-κB represents just one of the mechanisms required to ensure the transcriptional response is tightly regulated. The link between ubiquitination and the control of the NF-κB pathway is not new, however, previous work centred on the role of ubiquitination in NF-κB activation and particularly the ubiquitin triggered proteasomal degradation of the cytoplasmic IκB proteins. It is now apparent that ubiquitination and proteasomal degradation of NF-κB subunits represents a major limiting factor in the NF-κB transcriptional response. Post-translational modification of NF-κB is an intense area of research and while the predominant focus of much of the work to date has been on p65, information gathered from these studies will benefit our understanding of other NF-κB subunits.

Significant progress has been made in elucidating the mechanisms involved in the ubiquitination of p65 however there are a number of outstanding questions. Why does p65 require multiple E3 ligases? As described, several E3 ligases have been identified to act on p65 and from the available data these appear to regulate both overlapping and distinct NF-κB target genes. One explanation may be that different p65 E3 ligases have stimulus- or cell type specific functions. Multiple TLR and TNFR ligands induce p65 ubiquitination, however NF-κB target genes are differentially regulated by individual stimuli. It is conceivable therefore that ubiquitination of p65 is mediated by different E3 ligases dependent on the context of the signal or the cell type. Furthermore as p65 exists as part of both homo- and hetero-dimers, the possibility of dimer specific E3 ligases cannot be ignored. For example, in addition to p65, PPARγ also interacts with p50, however this is likely as part of a p65 heterodimer as interaction is dependent on p65. How does DNA binding trigger ubiquitination? What determines the specificity of the ubiquitin acceptor lysine? While it is tempting to speculate, it is clear there is yet much to discover about NF-κB ubiquitination and how individual subunits are regulated.

The UPS is an attractive target for the development of novel therapies for the treatment of many immunological diseases and cancer. However, the complexity of the system is such that establishment of selective inhibitors for clinical use is challenging and compared to kinase inhibitors, has been relatively unsuccessful. A promising strategy is the development of specific inhibitors of E3 ligase-substrate interactions [203]. Hundreds inhibitors of NF-κB activation have been identified but these are generally limited by having broad specificity [204]. Blocking the ubiquitination and degradation of specific NF-κB dimers, for example the repressive p50 homodimer, could be a useful tool not only to provide a greater insight into the role of ubiquitination in the regulation of transcriptional activity, but also development of NF-κB based therapeutic agents for inflammatory diseases. Alternatively, strategies aimed at preventing NF-κB deubiquitination may provide a more straightforward approach to target NF-κB transcriptional activity.

## Figures and Tables

**Table 1:** List of posttranslational modification associated with the ubiquitination of NF-κB subunits.

Subunit	Amino Acid	Enzyme/Stimuli	Modification	Function	Ref
p65	K28	PPARY	Ubiquitination	Degradation	[116]
	K62	ING4	Ubiquitination	Degradation	[95,96,117]
	K195	TNF	Ubiquitination	Degradation	[95,118]
	K56, K79, K123, K310, K315	Unknown	Ubiquitination	Unknown	[95,96]
	S468	TNF	Phosphorylation	Promotes ubiquitination	[102,103]
	T254/P255	Pin-1	Proline isomerization	Inhibits ubiquitination	[119]
p105	S927,S932	IKKβ	Phosphorylation	Recruits SCF^β−TrCP^	[120]
	Unknown	SCF^β−TrCP^	Ubiquitination	Degradation	[49]
	Multiple lysines	KPC1	Mono ubiquitination	Processing	[121]
p100	K855	SCF^β−TrCP^	Ubiquitination	Processing	[122]
	S870/S872	NIK/IKKα	Phosphorylation	Processing	[123,124]
	Unknown	SCF^Fbw7α^	Ubiquitination	Degradation	[125,126]
	S707	GSK3β	Phosphorylation	Promotes ubiquitination	[125,126]

## Abbreviations

The following abbreviations are used in this manuscript:

ARD	Ankyrin repeat domain
ATP	Adensoine triphosphate
BAG1	BCL2-associated athanogene 1
cIAP	Inhibitors of apoptosis proteins
COMMD1	Copper metabolism Murr1 domain-containing 1
CRL	Cullin-RING ligase
DUB	Deubiquitinase
E1	Ubiquitin-activating enzymes
E2	Ubiquitin-conjugating enzyme
E3	Ubiquitin ligase
GRR	Glycine rich region
HECT	Homology to E6AP C terminus
HOIL-1L	Hame-oxidized IRP2 ligase-1
HOIP	HOIL-1-interacting protein
IKK	IκB kinase
ING	Inhibitor of growth
IκB	Inhibitor of κB
LAP	Leukemia-associated protein
LPS	Lipopolysaccharide
LUBAC	Linear ubiquitination assembly complex
JAMM	JAB1/MPN/Mov34
NEMO	NF-kb essential modulator
NF-κB	Nuclear factor kappa b
OUT	Ovarian tumor proteases
PDLIM2	PDZ -LIM domain containing protein 2
PHD	Plant homeodomain
PMA	Phorbol 12-myristate 13-acetate
PPAR	Peroxisome proliferator activated receptor
RBR	RING-between-RING
RHD	REL homology domain
RING	Really interesting new gene
SCF	Skp1–Cullin1–F-box protein
SHARPIN	Shank1, a postsynaptic density-enriched protein
SOC1	Suppressor of cytokine signalling 1
TLR	Toll like receptor
TNFR	TNF receptor
UCH	Ubiquitin carboxy-terminal hydrolases
USP	Ubiquitin specific proteases
UPS	Ubiquitin proteasome system
